# Psychological Factors to Consider in Treating Patients with Acne Vulgaris

**DOI:** 10.3390/jcm15166347

**Published:** 2026-08-17

**Authors:** Julia Woźna, Paweł Pazdrowski, Natalia Welc, Anita Płócienniczak, Katarzyna Korecka, Ryszard Żaba, Andrzej Grzybowski, Ewa Mojs

**Affiliations:** 1 Department of Dermatology and Venereology, Poznan University of Medical Sciences, 60-355 Poznań, Poland; 2 Doctoral School, Poznan University of Medical Sciences, 61-701 Poznań, Poland; 3 Faculty of Medicine for Ms Anita Płócienniczak, Poznań University of Medical Sciences, 61-701 Poznań, Poland; 4 Department of Ophthalmology, University of Warmia and Mazury, 10-719 Olsztyn, Poland; 5 Institute for Research in Ophthalmology, Foundation for Ophthalmology Development, 61-553 Poznań, Poland; 6 Department of Clinical Psychology, Poznan University of Medical Sciences, 61-701 Poznań, Poland

**Keywords:** acne vulgaris, psychodermatology, psychological stress, quality of life, treatment adherence

## Abstract

Acne vulgaris is a chronic inflammatory skin disease associated with a substantial psychological burden that may extend beyond visible lesions and objective disease severity. Psychological factors such as stress, coping strategies, quality-of-life impairment, self-esteem, psychiatric symptoms, and treatment adherence are associated with disease perception, self-reported exacerbation, and treatment-related outcomes. This narrative review examines psychological stress and proposed biological mechanisms, coping strategies, treatment adherence, quality of life, self-esteem, sleep disturbance, and psychiatric comorbidity in people with acne. Relevant literature was identified through MEDLINE/PubMed, Scopus, Web of Science, and PsycINFO; the final literature search was conducted on 29 May 2026. Acne-specific studies were prioritized, with broader psychodermatology evidence used where acne-specific data were limited. The psychosocial burden of acne is individualized and only partly aligned with clinician-rated severity, supporting person-centered assessment. For each domain, the review outlines brief, commonly used screening instruments that may be feasible in routine dermatology. Incorporating psychological assessment and targeted supportive strategies into acne care may contribute to quality of life, adherence to therapy, and patient-centered care. The evidence is predominantly cross-sectional and derives from heterogeneous populations, instruments, and study designs, limiting causal inference.

## 1. Introduction

Acne vulgaris is a chronic inflammatory disease affecting the pilosebaceous unit and is among the most commonly treated skin conditions worldwide [1]. While it primarily occurs in adolescents, it can also impact adults, particularly women [2,3,4]. Acne prevalence is highest among teenagers, particularly in the 15–19 age group [5].

Although acne is not typically life-threatening, its clinical significance extends far beyond the presence of skin lesions. The burden of acne is multidimensional, encompassing not only physical symptoms but also emotional and social consequences that affect an individual’s quality of life [1,6]. The condition often arises during significant psychosocial changes, body-image development, and the formation of interpersonal relationships [4,7].

In recent years, there has been a growing focus on the psychological aspects of acne. Individuals with acne experience symptoms of anxiety, depression, and psychological stress significantly more frequently than the general population [8,9]. Despite this, the psychological impact of acne is often underestimated in everyday practice, even though it can drastically influence a person’s perception of the condition, social functioning, and expectations regarding treatment [2,4].

A crucial clinical issue is the disproportionate relationship between the psychological burden and the objective severity of skin lesions. Research on health-related quality of life indicates that acne can affect emotional and psychosocial domains, with patients reporting considerable distress even with mild skin changes [6,7]. Additionally, long-term consequences of acne, such as post-acne scars, can perpetuate psychological discomfort and diminish self-esteem, even after active inflammation has subsided [10].

The relationship between acne and mental health is likely bidirectional, but most evidence is cross-sectional or based on self-reported exacerbation and therefore supports association rather than causal direction. Stress, anxiety, and low mood may coincide with acne worsening, while visible lesions may contribute to poorer psychological well-being and quality of life [2,8,9]. This supports a broader, more individualized approach that extends beyond lesion counts [4,6]. In practice, psychosocial factors may be associated with adherence, satisfaction, and treatment experience, but they are not routinely assessed.

This paper aims to present the psychological factors associated with acne vulgaris, including stress, coping strategies, quality of life, self-esteem, co-existing mental disorders, and treatment adherence. The review is organized around three questions: (1) Which psychological factors are most consistently associated with acne-related burden? (2) Which brief instruments are feasible in routine dermatological practice? (3) When should dermatologists consider mental-health referral? It also describes brief, commonly used screening instruments that may support a holistic, patient-centered approach. The review covers adolescents and adults with acne and integrates several psychological domains with a practical clinical pathway rather than focusing on quality of life alone.

## 2. Materials and Methods

This article is a narrative review. It does not follow a systematic-review protocol. The aim was to synthesize, in a clinically oriented manner, the psychological domains most relevant to acne vulgaris and the brief instruments applicable to their assessment.

Relevant literature was identified by searching MEDLINE/PubMed, Scopus, Web of Science, and PsycINFO for records published up to 2026; the final literature search was conducted on 29 May 2026. Search terms combined disease concepts with psychological concepts, for example: (“acne” OR “acne vulgaris”) AND (“stress” OR “coping” OR “adherence” OR “compliance” OR “quality of life” OR “self-esteem” OR “sleep” OR “depression” OR “anxiety” OR “body dysmorphic disorder” OR “eating disorder” OR “suicide” OR “screening” OR “psychodermatology”). The complete search strategy is provided in Supplementary Material File S1. Reference lists of relevant articles were hand-searched to identify additional sources.

Eligible sources included observational studies (cross-sectional, cohort, case–control), clinical trials, validation studies of assessment instruments, qualitative studies, and prior reviews and meta-analyses. Publications in English were considered. Priority was given to studies conducted specifically in acne vulgaris; where acne-specific evidence was limited, evidence from other dermatological conditions (for example, psoriasis and atopic dermatitis) or from general psychiatric populations was included and is explicitly labeled in the text as indirect evidence.

Article screening and selection were performed by the authors, with disagreements resolved by discussion and consensus. The psychological domains were selected because they recur across the acne psychodermatology literature and map onto routine dermatological decisions; instruments were selected according to brevity, availability of validation data, and clinical feasibility. The final synthesis comprised 153 cited sources. Because identification, deduplication, and screening counts were not prospectively recorded for this narrative review, a PRISMA-style flow diagram and stage-specific counts could not be reconstructed reliably; no retrospective estimates are reported.

## 3. Stress in Acne Vulgaris

### 3.1. Psychological Stress in Acne Vulgaris

Depression, anxiety, and psychological stress are frequently reported among individuals with dermatological diseases, including acne vulgaris, and significantly impact psychosocial well-being and quality of life [11]. A recent meta-analysis by Salari et al., which included 113 studies, found pooled prevalence rates of 39% for stress, 27% for depression, and 29% for anxiety, respectively, across dermatological conditions [11]. When analyzed by diagnosis, acne vulgaris showed the highest prevalence of perceived stress, affecting 75.7% of patients. Although this figure should be interpreted as a pooled estimate from the included samples rather than as a universal prevalence, it underscores acne’s significant psychological impact among skin diseases [11].

Multiple observational studies indicate a temporal association between psychological stress and acne activity. Research in academic settings consistently shows that acne severity worsens during examination periods, supporting a connection between acute stress and disease exacerbation [12,13,14]. Among students, greater acne severity is associated with increased emotional and social burden and higher perceived stress [14,15,16]. Similar associations have been observed during large-scale societal stressors. Psychoemotional stress related to the war in Ukraine has been associated with self-reported acne worsening in over half of affected individuals, suggesting that extreme stress may influence disease activity [17].

Sebum secretion is generally elevated in acne [18], but available evidence does not clearly show that acute psychological stress directly increases sebum production. Survey-based studies have reported that approximately 50–80% of respondents perceived acne flares during periods of heightened stress, sometimes within days of stress exposure [19]. These estimates are study-specific and largely based on self-report rather than clinician-rated change; they therefore indicate a perceived temporal association, not proof that stress caused the flare. The same caution applies to examination-related and war-related studies that rely on self-reported worsening.

The relationship between acne and psychological distress is likely bidirectional. Individuals with acne more often report negative life events and higher levels of depressive and anxiety symptoms than healthy controls, suggesting that acne may function not only as a stress-responsive condition but also as a persistent psychosocial stressor [20,21]. This reciprocal interaction may contribute to a self-perpetuating cycle of psychological distress and disease exacerbation over time [19]. Acne-related stigma has been shown to independently predict impaired health-related quality of life, increased psychological distress, and somatic symptom burden, exceeding the effects of acne severity, lesion distribution, and treatment status [19,22].

Despite its high prevalence and clinical relevance, psychological support for acne remains markedly underutilized in routine care. Even as perceived stress increases with disease severity, only a minority of patients report being offered psychological screening or referral [23].

### 3.2. Biochemical Mechanisms Linking Stress and Acne Vulgaris

From a biochemical perspective, psychological stress affects acne through interconnected hormonal and immune pathways that link the body’s stress response to skin homeostasis [24]. Stress activates both central and skin-specific stress-response systems, including hypothalamic–pituitary–adrenal (HPA) axis signaling, which leads to the release of cortisol as part of the systemic stress response [25]. Stress also activates the sympathetic nervous system, increasing catecholamines such as adrenaline and noradrenaline, which may promote inflammation and worsen acne lesions [26]. Concurrently, stress-induced activation of HPA pathways involves proopiomelanocortin (POMC), a precursor protein mainly expressed in the pituitary and hypothalamus, and to a lesser extent in the skin. POMC is processed into biologically active peptides that participate in the regulation of sebaceous gland function and cutaneous immune responses [27]. Psychological stress may also alter tryptophan metabolism [28]. Cortisol and pro-inflammatory cytokines stimulate tryptophan 2,3-dioxygenase and indoleamine 2,3-dioxygenase, shifting tryptophan from serotonin synthesis toward the kynurenine pathway [29]. This increases the kynurenine/tryptophan ratio and generates metabolites linked to neuroinflammation, oxidative stress, and depressive symptoms [30]. Given the link between acne, anxiety, and depression, this pathway may represent a biochemical connection between psychological stress and acne exacerbation, although further research is needed to clarify its role.

Psychological stress is also associated with increased activity of neuropeptides involved in neurogenic inflammation. Substance P, a stress-related neuropeptide, is elevated in acne and is associated with inflammatory signaling and changes in sebaceous glands, potentially contributing to disease exacerbation [31,32]. In parallel, reduced serum levels of brain-derived neurotrophic factor (BDNF) are observed in acne patients and are associated with chronic stress, anxiety, and depressive symptoms, suggesting that BDNF may reflect sustained neurobiological stress responses in this population [33]. Higher levels of neurotensin, another stress-related neuropeptide, have been reported in acne, further supporting the connection between stress-related neuropeptide signaling and cutaneous inflammation [34].

Stress-induced skin responses also involve secretion of pro-inflammatory cytokines, particularly interleukin-1 (IL-1), IL-6, and IFN-γ [26,35]. Among these, IL-1α appears to play an important role in acne pathogenesis by keratinocyte activation, immune-cell recruitment, and cytokine signaling, thereby contributing to follicular inflammation and lesion development [36]. IL-1 may also increase keratinocyte 11β-hydroxysteroid dehydrogenase type 1, raising local cortisol levels in the skin [26]. This may contribute to comedogenesis [37,38].

Collectively, these biochemical changes influence inflammatory mediator release, sebaceous gland activity, and cutaneous nerve signaling within the pilosebaceous unit [39]. Stress-related neuroendocrine mediators act on multiple cellular targets—including sebocytes, keratinocytes, immune cells, and cutaneous nerves—supporting the concept that psychological stress amplifies cutaneous inflammation through coordinated neuroimmune–sebaceous interactions rather than isolated molecular events [40].

Beyond neuroendocrine and inflammatory pathways, psychological stress may influence acne by altering host–microbiome interactions [41]. Research indicates that individuals experiencing both acne and negative emotions have a unique skin microbiome profile, which may contribute to increased acne severity in this group [41]. *Cutibacterium*, *Acinetobacter*, and *Roseomonas* have been identified as key microbial taxa that may play a role in the pathogenesis of acne linked to emotional stress [41].

Studies indicate that stress-related catecholamines, such as epinephrine and norepinephrine, can alter *C. acnes* behavior without increasing its growth. Catecholamine exposure enhances biofilm formation in acne-associated strains and affects sebocyte function, indicating that *C. acnes* may serve as a biological interface between psychological stress and acne exacerbation [42]. Additionally, psychological stress alters host–microbiome interactions and triggers rapid biochemical responses in adult skin, reinforcing the concept of skin as an active, stress-responsive organ [43,44]. The principal stress-related mediators discussed above are summarized in Table 1, which distinguishes the type of supporting evidence (human, animal, in vitro, or mechanistic) and its certainty. Several of these—particularly the kynurenine pathway, BDNF, neurotensin, and microbiome findings—remain preliminary and should not yet be regarded as established acne mechanisms, nor do these biomarker associations currently have direct clinical utility.

### 3.3. Coping Strategies in Acne Vulgaris

Coping strategies are the cognitive and behavioral efforts individuals use to manage psychological stress and its emotional effects. As patients adjust to a chronic skin condition, they may gradually develop more effective coping mechanisms, which may also suggest greater psychological vulnerability in younger individuals [45]. Acne patients often experience psychosocial stressors such as facial visibility, perceived stigma, and fear of negative social evaluation. These factors can lead to maladaptive coping responses, including avoidance, emotional suppression, and social withdrawal [32,46]. Evidence shows a significantly higher prevalence of avoidance-oriented coping among individuals with acne [32,47]. Maladaptive coping may also encompass addictive behaviors, which have recently been reported at notable rates in patients with other chronic skin diseases, where a higher Dermatology Life Quality Index score was associated with several addictions [48]. Comparable data for acne vulgaris are currently lacking, and whether similar associations apply to this population remains to be established. In contrast, adaptive coping strategies such as problem-focused coping, active stress management, cognitive reappraisal, and seeking social support are linked to better emotional adjustment and greater psychological resilience [49]. Given the psychological burden of acne, assessing coping strategies and emotional functioning is clinically relevant. Providing psychological support and referrals as part of dermatological care, especially when significant distress is present, can help tailor stress-management approaches to individual needs and lifestyles [19,45,49].

Overall, these findings show that coping with stress is a key aspect of psychological well-being in acne vulgaris and support integrating stress-coping assessment and targeted psychological support into holistic acne management.

### 3.4. Clinical Tips and Tools

Addressing psychological stress in patients with acne vulgaris is clinically important, as stress not only contributes to disease exacerbation but also affects quality of life, treatment adherence, and long-term outcomes [50,51]. Coping responses influence how individuals adapt to stress; therefore, assessment of coping strategies may provide additional clinical insight. Early identification of patients experiencing significant psychological burden allows for timely supportive interventions, which have been shown to improve both psychological well-being and, in selected cases, acne severity [52,53].

In routine practice, stress and coping can be assessed using brief, commonly used self-report instruments. These tools allow clinicians to obtain a multidimensional overview of patients’ psychosocial functioning.

Direct stress assessment

- Perceived Stress Scale (PSS)—an instrument for assessing the degree to which situations in one’s life are perceived as stressful [54].
- Depression Anxiety Stress Scale–21 (DASS-21)—includes a dedicated stress subscale and allows simultaneous assessment of depression, anxiety, and stress symptoms [55].

Coping assessment

- Coping Inventory for Stressful Situations (CISS)—evaluates three major coping styles: task-oriented, emotion-oriented, and avoidance-oriented coping [56].
- Brief COPE (mini-COPE)—a brief multidimensional instrument assessing coping strategies such as active coping, acceptance, and avoidance [57]. It should be noted that most coping instruments (for example, the Brief COPE and CISS) were validated in general or mixed populations rather than specifically in acne, so their acne-specific performance is not established and results represent indirect evidence. Screening for coping has limited value unless it is linked to a response. When avoidance, social withdrawal, or appearance-related safety behaviors are identified, practical steps include brief validation and psychoeducation, encouragement of problem-focused strategies and social support, optimization of effective acne treatment to reduce the stressor itself, and referral for psychological support when distress is significant or when safety/checking behaviors suggest possible body dysmorphic disorder.

## 4. Adherence

Adherence and compliance are terms used to describe a patient’s behavior in relation to healthcare professionals’ recommendations [58]. Traditionally, the concept was centered on compliance, which reflects an asymmetrical relationship in which the physician provides instructions regarding diagnosis and treatment and the patient is expected to follow them as instructed [59]. In this model, the physician assumes a sole, authoritative role based on the belief that the healthcare professional knows best, both about the disease and its management [59,60]. In contrast, adherence represents a more collaborative and patient-centered approach. It describes a relationship in which the patient has greater autonomy in following medical recommendations [61]. The patient is actively involved in the decision-making process and retains the right to accept or refuse proposed tests or treatments [59]. In this model, the doctor–patient relationship is grounded in open communication, mutual respect, and shared responsibility. Better adherence is generally associated with improved acne outcomes, although the magnitude of benefit varies across regimens and studies [62].

Some reports describe very low adherence and others substantially higher levels [63,64]; these estimates are study-specific and are not directly comparable because populations, definitions, and measurement methods differ. More broadly, medication non-adherence has been associated with hospital admission and poorer control of chronic conditions [60,65], but estimates from general healthcare populations should not be assumed to apply directly to acne.

Associations between sociodemographic characteristics and adherence have been reported in dermatological populations, but findings are inconsistent and are not always acne-specific [58]. In one study regarding patients with psoriasis, women, employed participants, and married participants had higher reported adherence than their comparison groups [66]. Age-related findings are mixed: two reports described low adherence among children and adolescents with acne [67,68], another reported better adherence in younger patients [69], and reduced adherence in older adults may reflect cognitive impairment, polypharmacy, or sensory limitations [70]. Other studies found no significant age association [66,71]. Lifestyle associations have also been reported [58,66]; in one study, non-smokers and participants not reporting alcohol use had higher adherence than comparison groups [66]. Some of the studies cited concern various chronic dermatological conditions; however, the available data specifically addressing acne remain relatively limited. Therefore, further research is warranted to better explore this issue. Several of these sociodemographic findings derive from broader skin-disease or mixed dermatological populations rather than acne specifically and should be regarded as indirect evidence when applied to acne.

### 4.1. Determinants of Treatment Adherence

Adherence may be influenced by the prescribed treatment and its formulation [59]. Some studies suggest that adherence may differ between topical and oral therapies, although findings depend on the population and measurement method [66,71,72]. In an adolescent acne sample, adherence categories differed between oral and topical treatments, but the study-specific estimates should not be generalized beyond that setting [73]. Evidence from atopic dermatitis also suggests that formulation acceptability and ease of use may influence adherence; this is indirect evidence for acne [74]. A study comparing benzoyl peroxide formats found differences in reported convenience and comfort, suggesting that product acceptability may affect consistent use; however, the findings were specific to the formulations studied [75]. Treatment-related factors that may influence adherence include concerns and expectations, perceived need for treatment, dosing frequency, treatment duration, tolerability, time demands, previous experience, schedule, and cost [58,71]. Side effects, concerns about potent medications, and low perceived seriousness of acne have been reported as reasons for non-adherence [59]. Adherence may also differ between acute and chronic conditions, but estimates from broader dermatological populations should be applied cautiously to acne [59]. More complex regimens, more frequent dosing, longer treatment duration, and adverse events have been associated with lower adherence in some studies, although the magnitude and consistency of these associations vary [66]. Disease characteristics, including visibility and symptoms such as itching, may also influence adherence [59]. Financial barriers, including treatment cost and lack of insurance coverage, have also been reported as reasons for unfilled prescriptions [76]. Overall, reported adherence varies widely because studies use different definitions, formulations, treatment durations, populations, and measurement methods. The atopic dermatitis formulation data are indirect evidence and were not obtained in acne populations.

Mental health conditions are common in dermatological populations and may adversely affect treatment adherence [58,77,78]. Reduced adherence has been reported in individuals with symptoms of attention-deficit/hyperactivity disorder (ADHD) [72,79]. Symptoms such as forgetfulness and impulsivity may contribute to poorer adherence, while coexisting anxiety or depression may further complicate treatment routines [79]. Emotional-regulation difficulties among young people with acne may be associated with lower confidence in treatment and poorer adherence [73]. Depressive symptoms may also be associated with lower treatment satisfaction and less consistent adherence [80]. Adherence is an important contributor to treatment effectiveness. Further research is needed to clarify the factors that influence adherence and to identify effective, acne-specific support strategies.

### 4.2. Adherence Assessment

Below are several scales that may be helpful in a dermatology clinic when assessing the degree of adherence with medical recommendations. These are self-report instruments and therefore tend to overestimate adherence and are subject to recall and social-desirability bias; where feasible, they are best interpreted alongside objective measures (for example, pharmacy refill data or electronic monitoring).

- Morisky medication adherence scale-8 (MMAS-8)—a scale for assessing compliance with medical recommendations [73].
- Élaboration d’un outil d’évaluation de l’observance des traitements médicamenteux (ECOB)—a scale for assessing compliance with medical recommendations [63].
- Isotretinoin Hesitancy Scale (IHS)—a scale to assess beliefs and concerns related to the use of isotretinoin, potentially affecting adherence [81].

### 4.3. Clinical Strategies to Support Adherence

Patient education may support adherence [64,65,82] and can be delivered through short educational videos, accessible online materials, and informational leaflets [83]. Psychological support may also be considered to address emotional and behavioral barriers to treatment [65,73]. A collaborative patient–physician relationship may further support adherence [82], particularly when patients are empowered [59] and family members are involved when appropriate [59,84]. Written appointment schedules and clear medication instructions may also support adherence [59,85]. Reminder tools, such as mobile applications [84] and text or telephone reminders for medication use and follow-up, may be helpful [64,86]. Other potentially useful strategies include using combination medications and aligning dosing schedules with the patient’s routine [87], as well as simplifying regimens, considering once-daily treatment when appropriate, and developing individualized care plans [65,88]. One study suggested that adding an SPF 30 moisturizer and cleanser to benzoyl peroxide therapy may improve tolerability and satisfaction, but its effect on adherence should be interpreted cautiously [89]. Limited studies have explored desloratadine as an adjunct to isotretinoin, but the evidence is insufficient to support a general recommendation for improving adherence [90,91]. Discussing treatment costs and developing an alternative plan when a prescription cannot be filled may help reduce financial barriers. These strategies can be organized by barrier type: for practical barriers (forgetting, complex regimens), simplify the regimen and use reminders; for motivational barriers (low outcome expectancy), set realistic timelines and review progress; for emotional barriers (anxiety, low mood, frustration with slow response), address affect and consider psychological support; and for belief-related barriers (treatment concerns, isotretinoin hesitancy), provide balanced information and shared decision-making.

## 5. Quality of Life

Acne vulgaris is consistently reported in the literature as a dermatological condition with a substantial negative impact on quality of life, similarly to chronic conditions like epilepsy, asthma, diabetes, and some rheumatic diseases [92,93]. In acne vulgaris, quality of life is significantly impaired, reflecting the substantial psychosocial burden associated with the disease [94].

Studies demonstrate that acne affects multiple domains of daily functioning, including emotional well-being, social interactions, self-esteem, and interpersonal relationships [95,96,97]. Importantly, the burden on quality of life is not limited to active inflammatory lesions; acne scarring has been shown to exert a long-lasting psychosocial impact, contributing to persistent dissatisfaction with appearance, social avoidance, and emotional distress even after clinical resolution of acne [98].

Across diverse populations and age groups, impaired quality of life in acne patients has been associated with increased levels of anxiety, depressive symptoms, stress, and reduced self-confidence, with adolescents and young adults being particularly vulnerable due to heightened social sensitivity and body image concerns [99,100]. Several studies further indicate that perceived quality-of-life impairment does not always correlate with clinician-rated acne severity, underscoring the subjective and individualized nature of disease burden [101,102].

The consequences of impaired quality of life in acne extend beyond emotional distress. Reduced quality of life has been linked to social withdrawal, impaired academic or occupational performance, and reduced treatment adherence, potentially influencing long-term disease outcomes [103,104,105]. Recent studies continue to confirm that acne-related quality-of-life impairment remains highly prevalent despite advances in therapeutic options, highlighting an unmet need in comprehensive acne care [106,107,108].

Routine assessment of quality of life is increasingly recognized as an essential element of acne management, helping clinicians identify patients who may benefit from intensified dermatological or psychological interventions [109,110]. Failure to evaluate QoL may lead to underrecognition of patient burden and less patient-centered care. Appropriate treatment is therefore crucial, as untreated acne has been associated with long-term psychosocial consequences, including lower mental HRQoL and more frequent depressive symptoms in adulthood [111].

### Clinical Tools for Quality-of-Life Assessment in Acne

Incorporating quality-of-life assessment into routine acne care supports a holistic approach that addresses both physical manifestations and psychosocial consequences of the disease, ultimately improving patient satisfaction, adherence, and overall outcomes. Several instruments are recommended for use in clinical practice and research:

Quality-of-life assessment

- Dermatology Life Quality Index (DLQI)—the most widely used dermatology-specific instrument for assessing quality of life, including in patients with acne [112].
- Cardiff Acne Disability Index (CADI)—a brief acne-specific questionnaire focused on the emotional and social impact of acne [113].
- Acne-Specific Quality of Life Questionnaire (Acne-QoL)—assesses domains such as self-perception, social role, emotional role, and acne symptoms [114].
- Skindex—a comprehensive dermatology-specific quality-of-life instrument applicable to patients with acne and acne scarring [115].
- Quality of Life Relevance-Acne (QOLRA)—an acne-specific quality-of-life questionnaire developed to assess the psychosocial burden of acne [116].
- WHOQOL—a World Health Organization instrument for assessing general quality of life across multiple domains [117].

## 6. Self-Esteem

Acne vulgaris is associated with reduced self-esteem, highlighting the psychological burden of this visible and often stigmatizing condition [118]. Studies show that patients with acne, especially adolescents and young adults, have significantly lower self-esteem than healthy controls [119]. The psychosocial impact also tends to increase with acne severity [120].

A large population-based study of 3775 late adolescents from Norway, Germany, and the United States found that many individuals with acne reported negative self-evaluation. Feelings of worthlessness were reported by about 25% of females and 13% of males [120].

Adolescents with acne have lower self-esteem than controls, with the effect especially pronounced among females. Studies indicate that female sex, facial involvement, longer disease duration, and consequences such as post-inflammatory hyperpigmentation or scarring are linked to greater reductions in self-esteem [118,121,122].

Since self-esteem and personal identity develop during adolescence and young adulthood, a visible and potentially disfiguring skin disease can contribute to interpersonal difficulties and challenges in social, vocational, and sexual functioning, ultimately affecting psychosocial maturation [118,123].

Among adolescents, low self-esteem in those with acne is closely linked to symptoms of anxiety and depression, highlighting self-esteem as a key psychological mediator between acne and broader emotional distress [124]. Individuals with higher self-esteem generally experience better mental health, greater resilience, and improved quality of life compared to those with lower self-esteem [106].

These findings underscore reduced self-esteem as a key psychosocial burden of acne vulgaris. Because self-esteem is closely linked to emotional health, social interactions, and quality of life, it should be considered an essential component of holistic acne evaluation. Patients who perceive their treatment as beneficial typically report higher self-esteem, better quality of life, and lower anxiety than those who do not feel they have improved [118]. The association between acne severity and self-esteem is inconsistent across studies, with some finding little relationship between clinician-rated severity and self-esteem; findings should therefore be presented as variable rather than uniform. Sex-related differences (typically lower self-esteem in females) may reflect sociocultural and appearance-related pressures rather than an intrinsic biological difference. Self-esteem should not be treated as interchangeable with body image, depressive symptoms, or quality of life, even though these constructs overlap. Self-esteem may partly mediate the relationship between acne, perceived stigma, and depression, although this remains to be confirmed in longitudinal work.

### Clinical Tools Applicable in the Dermatology Clinic

In routine clinical practice, self-esteem in patients with acne vulgaris can be assessed using brief self-report instruments: Rosenberg Self-Esteem Scale scores should be interpreted as dimensional indicators of self-esteem, not as diagnostic thresholds.

Self-esteem assessment

- Rosenberg Self-Esteem Scale (RSES)—the most widely used instrument for assessing global self-esteem in adolescent and adult populations, including patients with acne [125].
- Coopersmith Self-Esteem Inventory (CSEI)—used mainly in adolescent populations to assess multidimensional self-esteem and self-concept [126].

## 7. Sleep Disturbances

Acne and sleep disturbances show a bidirectional relationship. Observational studies report associations between acne and poorer sleep quality, insomnia symptoms, anxiety, and depression; some also report correlations between acne severity and sleep impairment [127,128]. Because most studies are cross-sectional, these findings cannot determine whether acne contributes to sleep disturbance, sleep disturbance contributes to acne, or both reflect shared factors. Potential confounders include age, evening screen exposure, shift work, substance use, psychiatric symptoms, pruritus or pain, and medication effects.

Conversely, inadequate sleep is associated with higher acne prevalence or severity, possibly due to stress, hormonal changes, and inflammation, though causality remains unconfirmed [128,129]. Longer sleep duration has also been associated with lower acne risk in observational data [127].

Circadian factors may also play a role. Sleep–wake rhythms influence endocrine and immune regulation. Preliminary genetic studies suggested possible links between CLOCK gene variants and acne susceptibility [130]. Though evidence remains early, circadian rhythm gene polymorphisms may be associated with acne susceptibility, particularly in the context of night shift work [130].

The relationship between isotretinoin and sleep remains uncertain. Limited studies suggest sleep quality may change during treatment in some patients, but findings are inconsistent [131,132,133].

A recent study reported that depressive and anxiety symptoms partially mediated associations between sedentary behavior, later sleep timing, and acne [134]. This finding suggests that sleep health may be relevant to acne care, but it does not establish a causal pathway.

### Self-Report Instruments for Sleep Assessment

- Pittsburgh Sleep Quality Index (PSQI)—the most widely used instrument for assessing subjective sleep quality and sleep disturbances over the previous month [135]. The PSQI is a screening and symptom-quantification instrument, not a diagnostic tool for sleep disorders.
- Epworth Sleepiness Scale (ESS)—measures daytime sleepiness [136].
- Morningness–Eveningness Questionnaire (MEQ)—to determine circadian preference (chronotype), classifying individuals according to morning or evening tendencies in sleep–wake patterns [137]. Referral to a sleep specialist should be considered when a patient reports persistent insomnia despite sleep–hygiene measures, loud habitual snoring or witnessed apneas, excessive daytime sleepiness that impairs functioning or safety, or when a primary sleep disorder is otherwise suspected. Sleep complaints emerging with, or worsening during, systemic acne treatment also warrant closer evaluation.

## 8. Psychological Disorders

People living with acne frequently experience shame, embarrassment, self-stigma, reduced self-confidence, and avoidance of public exposure. This highlights the profound psychosocial impact of a visible skin disease [138]. These emotional responses are not limited to active lesions but may also persist in individuals with acne scars [139]. Evidence suggests that women with acne may feel more stigmatized and report higher anxiety than men [140]. Social perception studies demonstrate that individuals with acne are often viewed as less attractive and as having more negative social traits. In contrast, clear skin is linked to favorable impressions and social acceptance [141]. The instruments described in this section are screening and case-finding tools, not diagnostic tests. A positive or elevated score indicates the need for further clinical evaluation; it does not establish a psychiatric diagnosis, which requires assessment by an appropriately qualified professional. Dermatologists using these tools should interpret them as prompts for discussion and, where indicated, referral—not as a substitute for diagnostic assessment.

### 8.1. Depression, Anxiety, and Broader Mental Health Outcomes

A substantial body of research indicates that individuals with acne are more vulnerable to anxiety and depressive symptoms and may face a higher risk of suicidal ideation [109,142]. Acne is also associated with lower self-esteem and reduced psychological well-being [109,143]. Studies involving clinical and control populations have reported significantly higher anxiety levels among acne patients, particularly among women [144]. Mental health burden may also be influenced by symptoms accompanying acne, such as pruritus, which has been shown to correlate positively with anxiety and depression scores [145]. Moreover, disease severity and the need for systemic treatment have been linked to greater loneliness, discrimination, stress, and poorer mental health outcomes [146,147]. The relationship between isotretinoin and mood merits particular caution: although case reports and some studies have raised concern about depression and suicidal ideation, large pharmacoepidemiological studies and meta-analyses have not established a consistent causal link, and successful acne clearance may itself improve mood. Because acne severity is itself associated with depression and suicidality, isotretinoin-treated patients should be monitored for mood symptoms as a prudent clinical practice, without implying that the drug causes them.

### 8.2. Anhedonia, Alexithymia, and Sexual Well-Being

Beyond anxiety and depression, patients with acne may experience anhedonia and alexithymia. Anhedonia has been reported in a subset of patients, with some studies suggesting a higher prevalence among men and greater impairment in intimate social interactions [148]. Acne may also affect sexual functioning; in one study, more than half of female participants reported that acne negatively influenced sexual desire [149]. Additionally, alexithymia has been identified in a proportion of individuals with acne, suggesting difficulties in emotional processing [150].

### 8.3. Body Image, Eating Disorders, and Body Dysmorphic Disorder

Acne can significantly alter body perception and has been associated with disordered eating behaviors and body dysmorphic disorder (BDD) [147,151]. Patients with acne may exhibit greater dietary restriction and heightened concerns about weight and appearance than controls [147]. Studies using the Body Dysmorphic Disorder Questionnaire have reported BDD symptoms in a notable proportion of acne patients, particularly women, often accompanied by compulsive behaviors such as mirror checking and camouflage strategies [151]. BDD may further contribute to panic attacks, social anxiety, and maladaptive coping strategies [152].

### 8.4. Clinical Assessment and Practical Considerations

Given the wide range of psychological consequences associated with acne, several standardized tools may be useful in dermatology practice. These include FSFI (Female Sexual Function Index) for sexual dysfunction [149], EDE-Q (Eating Disorder Examination Questionnaire) for eating disorders [147], SHAPS (Snaith–Hamilton Pleasure Scale) and ACIPS (Anticipatory and Consummatory Interpersonal Pleasure Scale) for anhedonia [148], BDDQ (Body Dysmorphic Disorder Questionnaire) for body dysmorphic symptoms [152], Reynolds Adolescent Depression Scale (RADS) and Beck Adolescent Anxiety Scale (BAAS) for depressive and anxiety symptoms, respectively [109].

Studies emphasize that patients with acne and acne scars often report frustration, helplessness, and emotional distress. Building a trusting therapeutic relationship and addressing both dermatological and psychological needs are essential elements of effective care [143]. Importantly, the instruments needed (Table 2) are screening and symptom-monitoring tools. A positive or elevated score indicates that further evaluation is warranted; it does not establish a psychiatric diagnosis. Diagnosis of depression, anxiety, body dysmorphic disorder, an eating disorder, a sleep disorder, or any other mental disorder requires clinical assessment by an appropriately trained professional. Questionnaire scores should never be used in isolation to diagnose, and cut-offs are dimensional indicators rather than diagnostic thresholds.

### 8.5. A Stepped Screening-And-Referral Pathway

The following pragmatic, stepped pathway integrates the elements above. It is intended to support, not replace, clinical judgment, and at no step does a questionnaire score substitute for clinical assessment:

1. Assess dermatological severity and its distribution, including scarring, as part of routine acne care.
2. Ask briefly about psychosocial impact and administer one short acne-specific quality-of-life measure (for example, CADI or Acne-QoL) to gauge burden.
3. When distress, disproportionate appearance concern, low mood, marked anxiety, disordered eating, or poor sleep is evident, add the relevant brief screen, recognizing that these screens are intended to monitor symptoms rather than diagnose conditions.
4. Interpret positive screens clinically: confirm findings through discussion, consider confounders, and decide on management rather than acting on a score alone.
5. Escalate according to urgency: immediate assessment or emergency referral for suicidal intent, plan, or recent self-harm; urgent psychiatric referral for severe depression or anxiety, suspected BDD, or a suspected eating disorder; and routine referral or shared care for persistent distress, low self-esteem, or sleep disorder not responding to first-line measures.

## 9. Discussion

### 9.1. Principal Findings

This review consolidates evidence that acne vulgaris is consistently associated with psychological burden across several domains—stress, coping, adherence, quality of life, self-esteem, sleep, and psychiatric symptoms—and that this burden is often only partially aligned with clinician-rated severity. The strongest and most consistent associations concern impaired quality of life and elevated depressive and anxiety symptoms; other domains, particularly candidate biomarkers, microbiome changes, anhedonia, alexithymia, and sleep, rest on more limited or preliminary evidence.

### 9.2. Interpretation and Strength of Evidence

The relationship between acne and mental health is best interpreted as bidirectional and multifactorial, but the underlying evidence is dominated by cross-sectional designs, heterogeneous populations, and varied measures, so causal direction generally cannot be established. Associations for quality of life and for depressive and anxiety symptoms are relatively robust and replicated; proposed biological mechanisms are based on largely mechanistic, animal, or small-sample human studies and currently lack clinical utility as biomarkers. A recurring methodological issue is the conflation of symptom scores with diagnosed disorders: most dermatological studies measure the former, which supports screening but not diagnosis.

### 9.3. Clinical Implications

For practice, the implication is that dermatologists can feasibly identify psychosocial burden using brief instruments (Table 2) and act on it through education, shared decision-making, adherence support, and timely referral, without themselves conducting formal psychiatric assessment. Because reported adherence varies widely and is shaped by modifiable barriers, matching support to the specific barrier is likely to be more effective than generic advice. Screening should be coupled in advance with a clear referral pathway, especially for suicidality, severe mood or anxiety symptoms, suspected BDD, and suspected eating disorders. A recurring interpretive point across these domains is that improvement in psychological symptoms (for example, distress, anxiety, or quality of life) should be distinguished from improvement in acne severity: an intervention may benefit one without necessarily changing the other, and the two outcomes should be reported and evaluated separately rather than conflated.

### 9.4. Limitations

This is a narrative rather than a systematic review: study selection was not governed by a pre-registered protocol, no formal risk-of-bias appraisal or meta-analysis was performed, and the restriction to English-language sources introduces potential selection and language bias. Some conclusions rely on indirect evidence extrapolated from psoriasis, atopic dermatitis, or mixed dermatological and psychiatric populations, which may not generalize to acne. Publication bias and heterogeneity in instruments and cut-offs further limit comparability across studies.

### 9.5. Research Priorities

Priorities for future work include prospective and longitudinal studies to clarify the direction of the acne–distress relationship; interventional trials testing whether systematic psychological screening and support improve adherence, quality of life, and dermatological outcomes; validation of brief instruments and cut-offs specifically in acne populations across ages and cultures; adequately powered studies of candidate biomarkers and the skin microbiome; and rigorous, confounder-adjusted evaluation of isotretinoin in relation to mood and sleep.

## 10. Conclusions

Acne vulgaris is a chronic inflammatory skin disease associated with a significant psychological burden beyond visible symptoms. Stress, maladaptive coping, impaired quality of life, low self-esteem, psychiatric comorbidities, and poor treatment adherence can all affect disease perception, exacerbation, and long-term outcomes. Psychological assessment is clinically important for identifying patients at risk of distress and psychosocial challenges that may hinder treatment outcomes [50,51]. Evidence suggests that psychological interventions, such as cognitive–behavioral therapy and structured stress-management programs, may reduce stress and negative affect; their effect on acne severity itself is less certain and should be distinguished from their effect on psychological symptoms [50,53]. Therefore, optimal acne care should integrate standard dermatological treatment with a psychosomatic approach, including stress management, relaxation, education, and therapy for psychiatric comorbidities [52,97,153]. Incorporating psychological assessment and targeted supportive strategies into routine practice may contribute to improvements in quality of life, adherence to therapy, and patient-centered care. Finally, it should be emphasized that the instruments discussed here are screening and monitoring tools that support case identification; they do not establish a psychiatric diagnosis, which remains a clinical determination.

## Figures and Tables

**Table 1:** Proposed biological factors linking psychological stress and acne vulgaris. Several cited sources are not acne-specific: references [25,26,27,35,43] are general psychodermatology, skin-stress or skin-microbiome reviews, and references [28,30] derive from psychiatric and general tryptophan-metabolism research rather than acne studies. The acne relevance of these mediators is therefore inferential and should be interpreted as preliminary.

Mediator/Pathway	Change Reported in Acne or Under Stress	Proposed Role
HPA axis/cortisol [25,26]	Increased cortisol with stress; local cutaneous cortisol via 11β-HSD1	Systemic and local stress signaling affecting sebaceous and immune activity
Sympathetic catecholamines (adrenaline, noradrenaline) [26,42]	Elevated with stress	Pro-inflammatory; enhance *C. acnes* biofilm formation
POMC-derived peptides [27]	Stress-induced processing in pituitary/skin	Regulation of sebaceous function and cutaneous immunity
Substance P [31,32]	Elevated in acne	Neurogenic inflammation; sebaceous changes
Tryptophan–kynurenine pathway [28,30]	Shift from serotonin toward kynurenine under stress	Links neuroinflammation and depressive symptoms to acne
BDNF [33]	Reduced serum levels in acne	Marker of sustained neurobiological stress response
Neurotensin [34]	Higher levels reported in acne	Stress-related neuropeptide signaling in inflammation
Pro-inflammatory cytokines (IL-1α, IL-6, IFN-γ) [35,36]	Elevated	Follicular inflammation, comedogenesis (IL-1α)
Skin microbiome (*Cutibacterium/C. acnes, Acinetobacter, Roseomonas*) [41,42,43]	Altered taxa in acne with negative emotions; catecholamine-driven biofilm	Candidate stress–skin interface

**Table 2:** Brief screening and assessment instruments relevant to psychodermatological care in acne. Item counts, administration times, and cut-offs are approximate and depend on the version and validated translation used; users must confirm current licensing terms before clinical or research use. None of these instruments provides a psychiatric diagnosis.

Instrument (Abbrev.)	Construct/Domain	Approx. Time	Age Range	Suggested Clinical Use
Perceived Stress Scale (PSS-10/-14/-4) [53]	Perceived stress	2–5 min	Adolescent–adult	Gauge stress burden when stress-related flares suspected
DASS-21 [54]	Depression, anxiety, stress (subscales)	3–5 min	Adolescent–adult	Broad initial screen across three domains
Brief COPE (mini-COPE) [56]	Coping styles	5–10 min	Adolescent–adult	Identify maladaptive coping (avoidance, denial)
Dermatology Life Quality Index (DLQI) [111]	Dermatology-specific QoL	1–2 min	≥16 y (CDLQI for children)	General dermatology QoL; not acne-specific
Cardiff Acne Disability Index (CADI) [112]	Acne-specific disability	1–2 min	Adolescent–young adult	Preferred brief acne-specific measure
Acne-QoL [113]	Acne-specific QoL (4 domains)	3–5 min	Adolescent–adult	Acne-specific QoL when more detail needed
Skindex (-29/-16) [114]	Dermatology-specific QoL	3–5 min	Adult	Detailed dermatology QoL incl. scarring
Rosenberg Self-Esteem Scale (RSES) [124]	Global self-esteem	2–3 min	Adolescent–adult	Flag low self-esteem for support/monitoring
Pittsburgh Sleep Quality Index (PSQI) [134]	Subjective sleep quality (past month)	5–10 min	Adolescent–adult	Screen sleep quality; refer if disorder suspected
Body Dysmorphic Disorder Questionnaire (BDDQ) [151]	BDD symptoms (screen)	2–3 min	Adolescent–adult	Screen when concern is disproportionate to findings
EDE-Q [146]	Eating-disorder symptoms	10–15 min	Adolescent–adult	Screen disordered eating when suspected

Note: instrument specifications and cut-offs derive largely from general validation sources rather than acne populations. References [53,54,56,124,134] (PSS, DASS-21, Brief COPE, RSES, PSQI) were validated in general or mixed clinical samples, and references [111,114] (DLQI, Skindex) are dermatology-wide rather than acne-specific; only the CADI, Acne-QoL, BDDQ and EDE-Q entries are cited to acne-focused studies.

## Data Availability

No new data were created or analyzed in this study. Data sharing is not applicable to this article.

## Supplementary Materials

The following supporting information can be downloaded at https://www.mdpi.com/article/10.3390/jcm15166347/s1: Supplementary Material File S1: Full electronic search.
