# Supplementary material for: Psychological Factors to Consider in Treating Patients with Acne Vulgaris
Source: J Clin Med. 2026 Aug 17;15(16):6347. doi: 10.3390/jcm15166347 (PMC13513615; doi:10.3390/jcm15166347)
Supplement: Supplementary file 1 [file jcm-15-06347-s001.zip › jcm-4454088-supplementary.pdf]

Supplementary Material S1: Full electronic search strategy

("Acne Vulgaris"[Mesh] OR "acne vulgaris"[Title/Abstract] OR acne[Title/Abstract]) AND ("Stress, Psychological"[Mesh] OR stress*[Title/Abstract] OR "Coping Skills"[Mesh] OR coping[Title/Abstract] OR "Medication Adherence"[Mesh] OR "Patient Compliance"[Mesh] OR adherence[Title/Abstract] OR compliance[Title/Abstract] OR nonadherence[Title/Abstract] OR "non-adherence"[Title/Abstract] OR "Quality of Life"[Mesh] OR "quality of life"[Title/Abstract] OR "health-related quality of life"[Title/Abstract] OR HRQoL[Title/Abstract] OR "Self Concept"[Mesh] OR "self-esteem"[Title/Abstract] OR "self esteem"[Title/Abstract] OR "self-confidence"[Title/Abstract] OR "self confidence"[Title/Abstract] OR "Sleep"[Mesh] OR "Sleep Wake Disorders"[Mesh] OR sleep[Title/Abstract] OR insomnia[Title/Abstract] OR circadian[Title/Abstract] OR chronotype[Title/Abstract] OR "Depression"[Mesh] OR depress*[Title/Abstract] OR "Anxiety"[Mesh] OR anxi*[Title/Abstract] OR "Mental Disorders"[Mesh] OR psychiatr*[Title/Abstract] OR "mental health"[Title/Abstract] OR "psychological distress"[Title/Abstract] OR "Suicidal Ideation"[Mesh] OR "Suicide"[Mesh] OR suicid*[Title/Abstract] OR "self-harm"[Title/Abstract] OR "self harm"[Title/Abstract] OR "Body Dysmorphic Disorders"[Mesh] OR "body dysmorphic disorder"[Title/Abstract] OR dysmorphophobi*[Title/Abstract] OR "Body Image"[Mesh] OR "body image"[Title/Abstract] OR "Feeding and Eating Disorders"[Mesh] OR "eating disorder"[Title/Abstract] OR "eating disorders"[Title/Abstract] OR "disordered eating"[Title/Abstract] OR "Social Stigma"[Mesh] OR stigma*[Title/Abstract] OR anhedonia[Title/Abstract] OR alexithymia[Title/Abstract] OR "sexual function"[Title/Abstract] OR "sexual functioning"[Title/Abstract] OR "sexual dysfunction"[Title/Abstract] OR psychodermatolog*[Title/Abstract] OR screening[Title/Abstract] OR questionnaire*[Title/Abstract] OR instrument*[Title/Abstract] OR scale*[Title/Abstract] OR assessment[Title/Abstract])
